# Supplementary material for: Accelerating cell culture media development using Bayesian optimization-based iterative experimental design
Source: Nat Commun. 2025 Jul 1;16:6055. doi: 10.1038/s41467-025-61113-5 (PMC12218302; doi:10.1038/s41467-025-61113-5)
Supplement: Supplementary file 1 — Supplementary Information [file 41467_2025_61113_MOESM1_ESM.pdf]

Supplementary Information

1. Number of experiments formulae and calculations

Full screen considers a naïve grid search approach, used only to provide an estimate for the spread of the design space. Other DoEs considered include, full factorial design, fractional factorial design, central composite design (CCD), Frac CCD that considers a fractional factorial design instead of a full factorial design, Box Behnken Desing (BBD), and One-Factor-at-a-time (OFAT).

1.1. Cytokine Optimization for maintaining PBMC homeostasis

Continuous Design factors (k) = 8
Where relevant, $(levels)$ = 10

Table S1: Calculation for the number of experiments for the different designs of experiments strategy considering the PBMC cytokine optimization case study. k is the number of factors and $(levels)$ indicates the number of levels considered.

Design Method	Formulae	Number of experiments
**Full Screen**	$(levels)^k$	10^8
**Full Factorial – 2level (FF2)**	2^k	256
**Fractional Factorial (FracFac)**	2^{k-1}	128
**CCD**	$2^k + 2k + 1$	273
**Frac CCD**	$2^{k-1} + 2k + 1$	145
**BBD**	$2.k.(k-1) + 1$	113
**OFAT**	$k\,.(levels)$	80

1.2. Carbon source optimization for K.*phaffii* cultivation

Continuous Design factors (k) = 3
Categorical Design factors level = 1 categorical variable/19 categories
Where relevant, $(levels)$ = 10 for continuous factors

Since the standard DoEs are not equipped to automatically design for categorical variables, the modified calculation assumes that the continuous design is repeated for each category separately. This is made because there are no known connections or mapping between categories, each category represents a different hyperplane in the cuboidal design space. Additionally, since the categorical variable is linked to 1 of the continuous variables, the estimate for the OFAT is modified accordingly.
Finally, an estimate is added for a potential resource-conservation DoE. Here, we assume that a basic OFAT initial screen is performed for the 19 different categorical variables (different carbon sources here), requiring 38 experiments. Subsequently subset of relevant/influential categories are selected. This was assumed to be 10 based on in-house transcriptomics data which suggests 10 different clusters among the considered carbon sources. Following this, a CCD is performed for only the selected 10 categories.

Table S2: Calculation for the number of experiments for the different designs of experiments strategy considering the carbon source optimization case study. k is the number of continuous factors and $(levels)$ indicates the number of levels considered for continuous factors.

Design Method	Formulae	Number of experiments
**Full Screen**	$19 * (levels)^k$	19000
**Full Factorial – 3level (FF3)**	$19 * 3^k$	513
**Full Factorial – 2level (FF2)**	$19 * 2^k$	152
**Fractional Factorial (FracFac)**	$19 * 2^{k-1}$	76
**CCD**	$19 * (2^k + 2k + 1)$	285
**Frac CCD**	$19 * (2^{k-1} + 2k + 1)$	209
**BBD**	$19 * (2.k.(k-1)+1)$	247
**OFAT**	$19 * (k.(levels))$<br>19*10 + 2*10	210
**Prescreen + CCD**	19*2 +<br>$10 * (2^k + 2k + 1)$	188

1.3. Additive optimization for K.*phaffii* cultivation

Calculations follow a similar rationale as Section 1.3.

Continuous Design factors (k) = 9
Categorical Design factors level = 1 categorical variable/19 categories
Where relevant, $(levels)$ = 10 for continuous factors

Table S3: Calculation for the number of experiments for the different designs of experiments strategy considering the carbon source optimization case study. k is the number of continuous factors and $(levels)$ indicates the number of levels considered for continuous factors.

Design Method	Formulae	Number of experiments
**Full Screen**	$19 * (levels)^k$	$19 * 10^8$
**Full Factorial – 3level (FF3)**	$19 * 3^k$	124659
**Full Factorial – 2level (FF2)**	$19 * 2^k$	4864
**Fractional Factorial (FracFac)**	$19 * 2^{k-1}$	2432
**CCD**	$19 * (2^k + 2k + 1)$	5187
**Frac CCD**	$19 * (2^{k-1} + 2k + 1)$	2755
**BBD**	$19 * (2.k.(k-1)+1)$	2147
**OFAT**	$19 * (k.(levels))$<br>19*10 + 8*10	270
**Prescreen + Frac CCD**	19*2 +<br>$10 * (2^{k-1} + 2k + 1)$	1468

2. Retrospective Kernel Impact Evaluation

2.1. One hot encoding kernel and the categorical overlap kernel

To retrospectively evaluate the impact of the kernel choice against the routine one hot encoding (OHE) approach, we used the data collected in this work and performed 50 random train–test splits of the data (80-20%). We trained the model on 80% data tested it on the remaining 20% and computed the root mean squared error in prediction (RMSEP), providing a distribution of 50 RMSEP.

$$\text{RMSEP} = \sqrt{\sum_{t=1}^{Ntest} \frac{\left(y_t^{obs} - y_t^{pred}\right)^2}{N}} \qquad \text{(Eq. S1)}$$

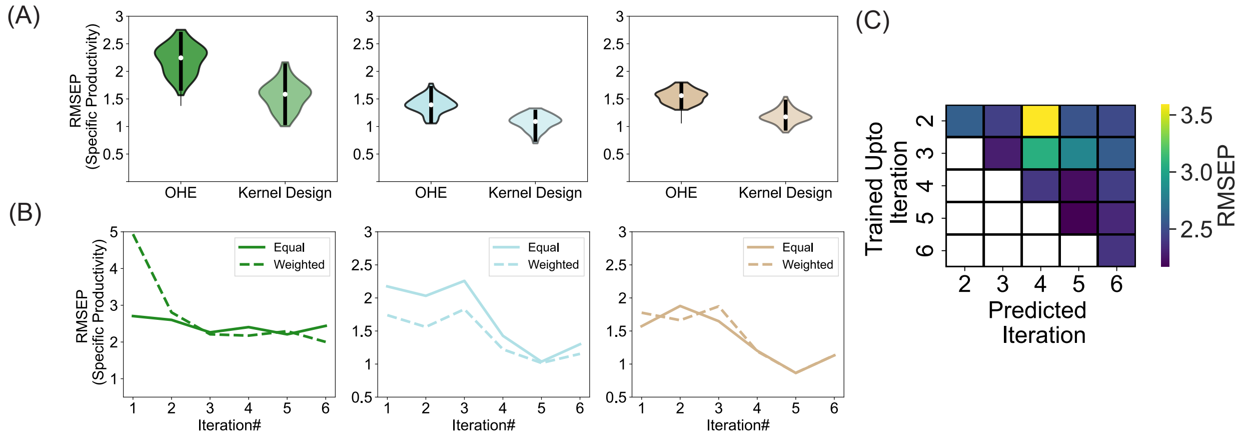

Fig S1: (A) Comparison of the RMSEP made by GP built using the OHE and designed kernel (B) Evolution of the predictive capability of the model in each iteration (C) Accuracy of the model in each iteration (x-axis) when accounting for progressively smaller subsets of data.

2.2. Matern32 and Matern52 kernel

For the PBMC basal media blend optimization case study, an a-posteriori analysis was performed to compare the performance of the Matern kernel with $\nu = 1.5$ (Matern32) and $\nu = 2.5$ (Matern52) to validate the choice of the kernel. We observed that the predictive performance of the GP model with both kernels was observed to be comparable.

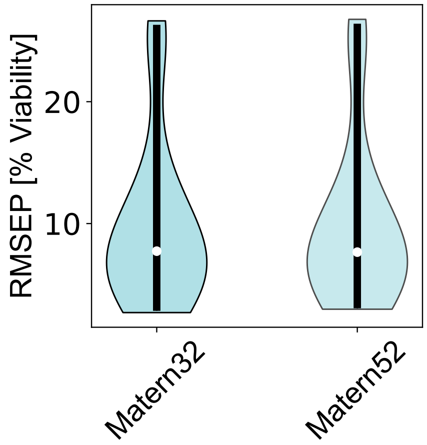

Fig S2: Distribution of the RMSEP for GPs trained on 50 different train test splits using the Matern kernel with ν = 1.5 (Matern32), and ν = 2.5 (Matern52)

2.3. Initialization of kernel hyperparameters

For the K.*phaffii* cultivation use cases, both the carbon source optimization (Fig S3A) and the additive optimization with 9 factors (Fig S3B), we performed a retrospective analysis to test the predictive capabilities as a function of hyperparameters' initialization. 19 different random initializations (blue violin plots) were tested and compared against the currently used parameter initialization (red violin plot). 50 different train-test splits were tested to assess the predictive capabilities, quantified using RMSEP.

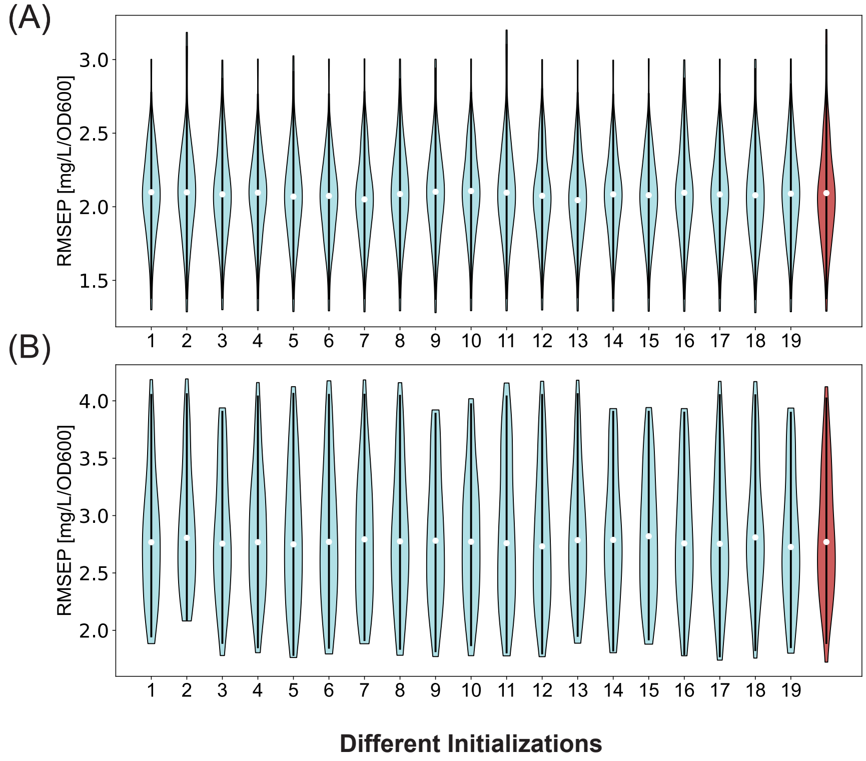

Fig S3: Comparison of the RMSEP made by GP kernels initialized with different random combinations of hyperparameters for (A) carbon source optimization of K.*phaffii* cultivation for recombinant production of RBDJ, and (B) additive optimization considering the 9 factors.

3. Converge Calculations

In this case, the model convergence was tracked through two approaches (i) the root mean squared error (RMSEP) and weighted RMSEP (ii) Progressive model prediction (Methods).

To check for convergence and the stopping criteria, we monitored the RMSEP and the weighted RMSEP for each iteration. In other words, during every iteration, we recorded the model prediction and compared it with the experimental observation obtained after performing the experiment. When the RMSEP made in two consecutive rounds is not significantly different it was determined as the indication of model convergence. To compute the weighted RMSEP the prediction with higher specific productivities (SP_t) were weighted higher by computing the weights as indicated in (Eq. S3).

$$RMSEP_{weighted} = \sqrt{\frac{\sum_{t=1}^{Ntest} w_{t*}\left(y_t^{obs} - y_t^{pred}\right)^2}{\sum_{t=1}^{Ntest} w_t}} \quad \text{(Eq. S2)}$$

$$w_t = \frac{SP_t}{\max(SP)} \quad \text{(Eq. S3)}$$

In addition, in every iteration, we also used a progressive model prediction approach. For instance, to predict the outcome of experiments in iteration 5, we would use models trained using data up to iterations 5, 4, and 3. We monitor the RMSEP made by each of these models trained on progressively smaller subsets of data. The point at which adding future iteration data is not crucial to the predictive performance was considered the checkpoint for model convergence.

The RMSEP (“Equal”, Fig. S1 B) provides an estimate of the overall model accuracy, while the weighted RMSEP skews the metric to provide higher weightage to desired targets (higher specific productivities) in the calculation. As the iterations progress, both RMSEP and weighted RMSEP reduce indicating the improvement in the model accuracy with iterations 5 and 6 having comparable errors (Fig. S1 B). Also, from iteration 4 onwards there is an agreement between both metrics. Combined with the observation made about the design space (Fig. 5A), this can be attributed to the fact that the algorithm is predominantly exploiting the design space. With the progressive model prediction approach (Fig. S1 C), we also see that the RMSEP for a model trained with data up to iteration 4 performs comparably to a model trained with data up to iteration 6 thus providing an additional indication that the model is converged.

4. Design space coverage of the PBMC blending: Univariate factor evolution

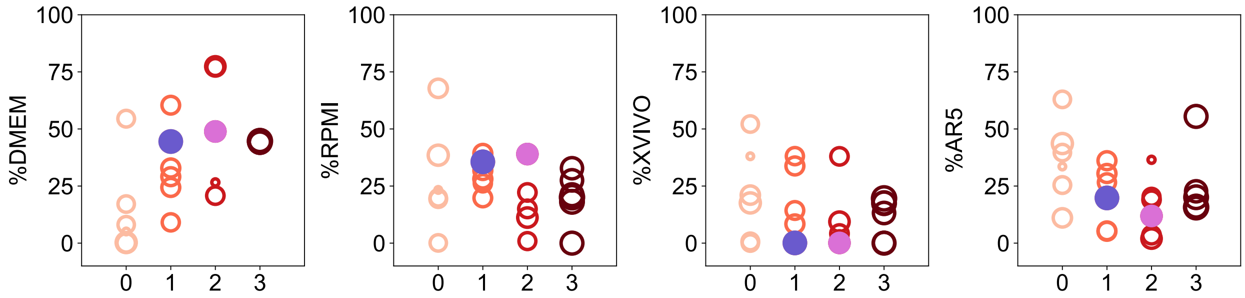

Fig S4: Evolution of the individual factors in the different iterations. The size of the marker indicates the corresponding average viabilities. The progressively increasing shades of red correspond to the increasing iteration number, purple color represent Media #12 and pink color correspond to Media #15, in line with Fig. 4. Source data are provided in Source Data.xlsx file

5. Design space coverage of the carbon source optimization: Univariate factor evolution

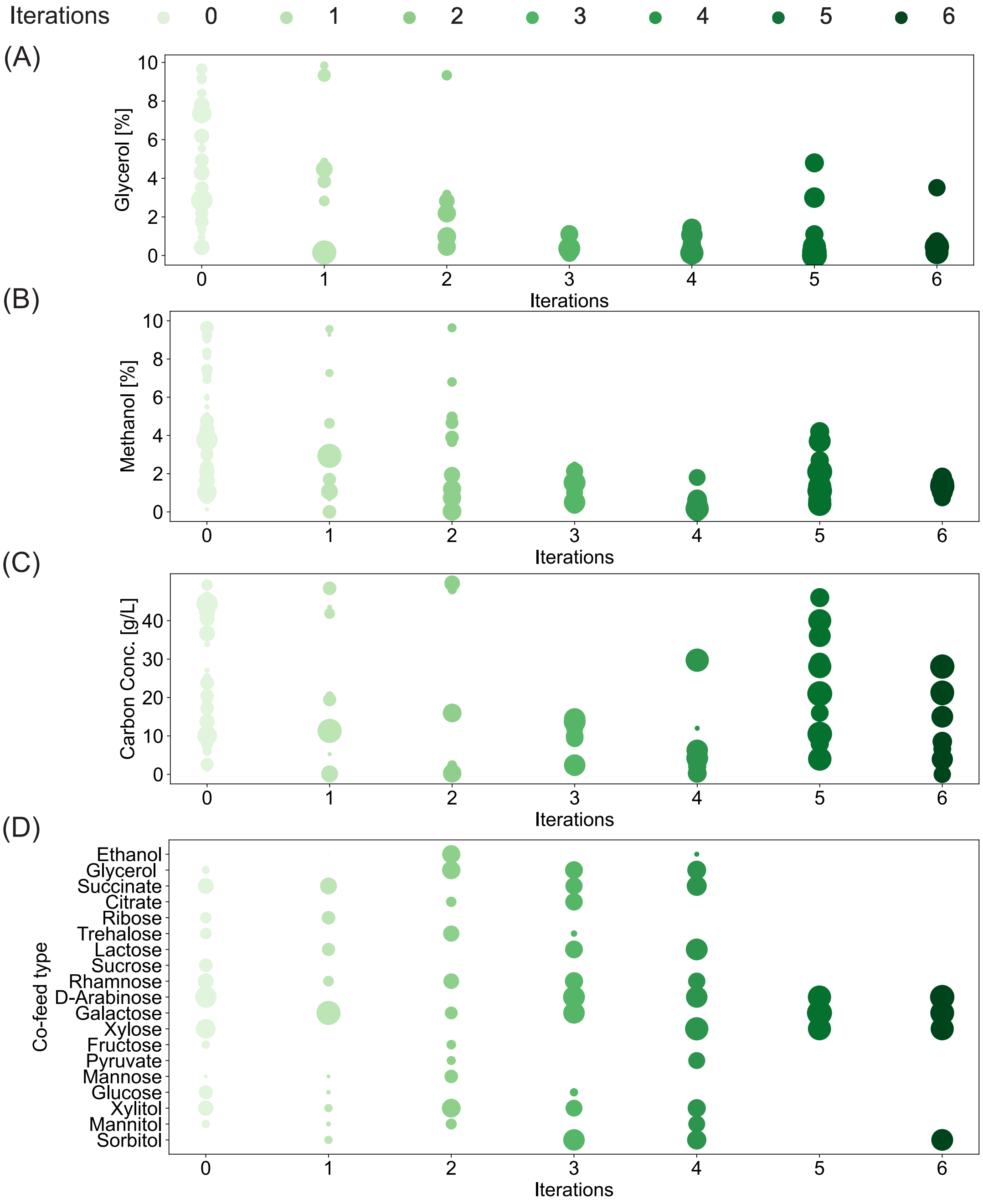

Fig S5: Evolution of the individual factors in the different iterations: (A) Glycerol, (B) Methanol, (C) Carbon source concentration, and (D) Carbon source feed type. The size of the marker indicates the corresponding specific productivity. Source data are provided in Source Data.xlsx file

6. Design space coverage of the extended design space considering the 9 factors

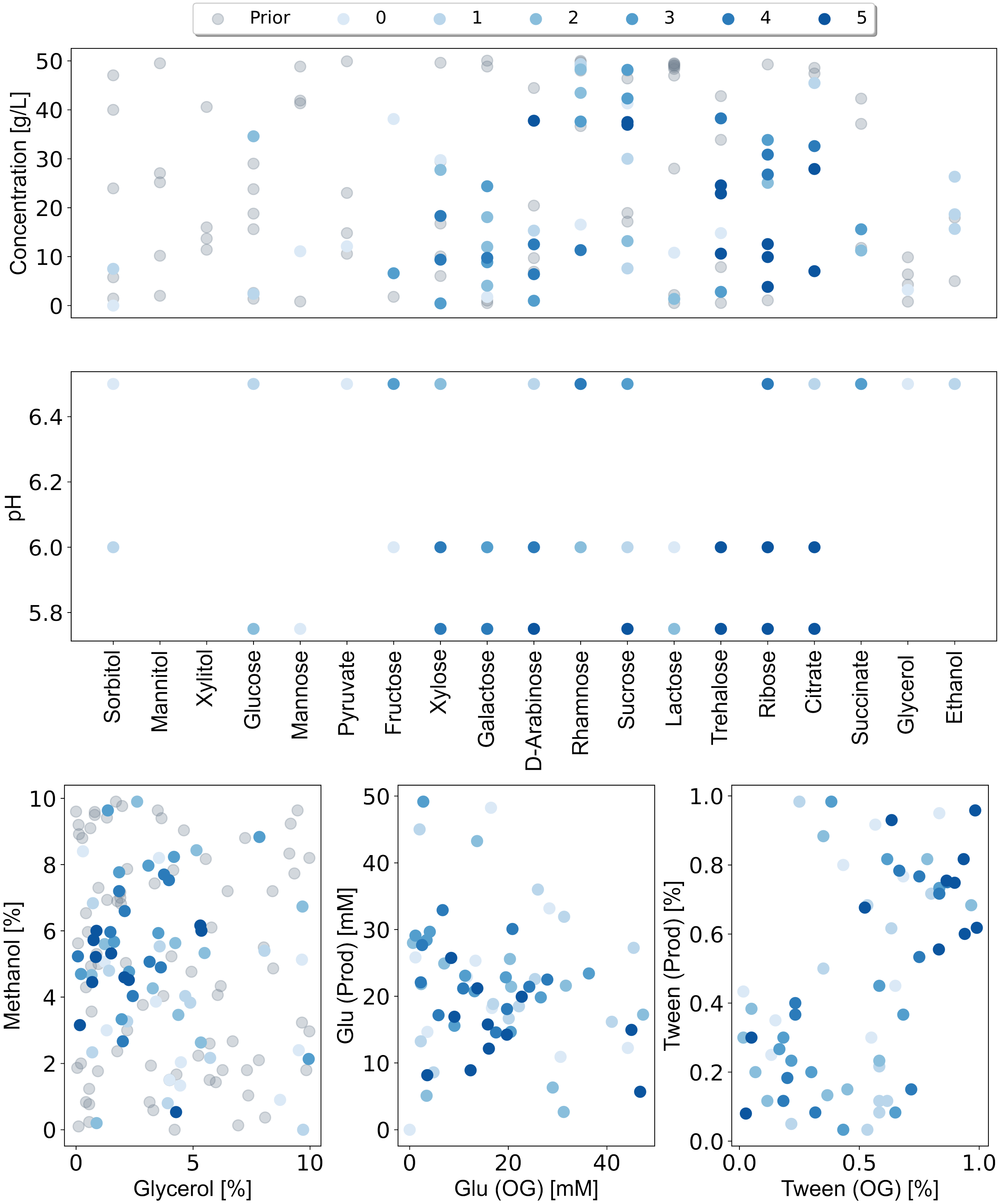

Fig S6: Evolution of the location of the experiments in the design space in the different iterations indicated through pairwise plots of the design factors. Source data are provided in Source Data.xlsx file.

7. Flow Gating Strategy

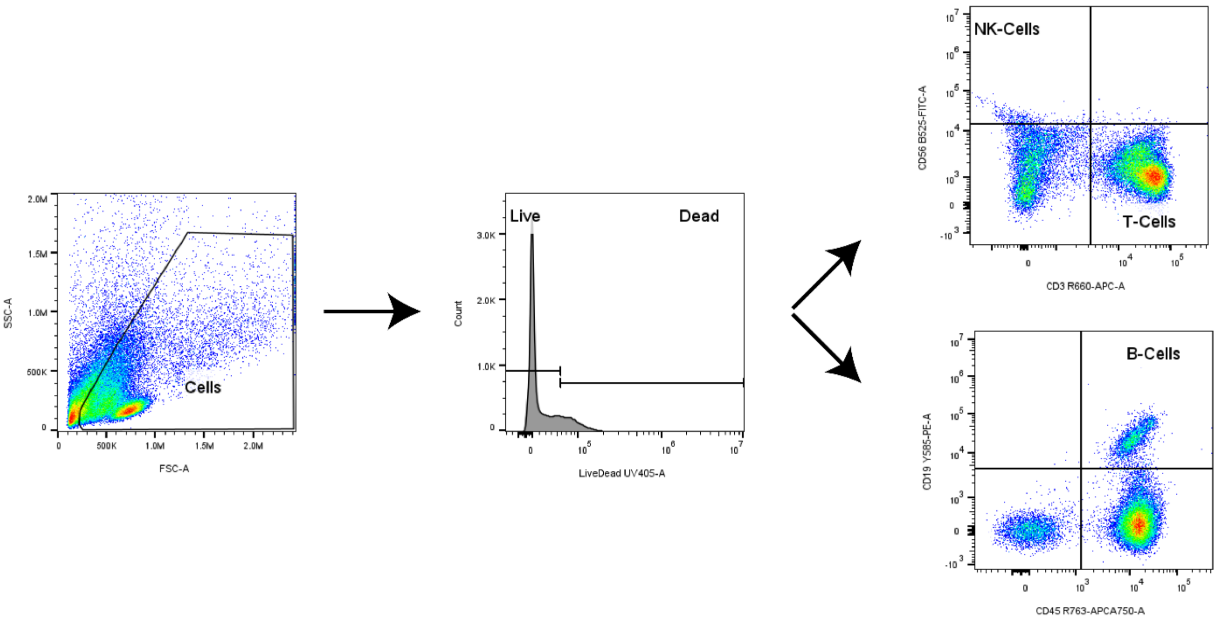

Fig S7: Flow cytometry gating strategy for cell population identification. Prior to sample acquisition, the flow cytometer was calibrated using fluorophore-labeled reference beads matched to the emission spectra of the experimental antibody panel. This calibration step was used to optimize voltage gain settings and establish fluorescence intensity cut-offs for positive and negative populations. Unlabeled control cells were subsequently analyzed to assess background autofluorescence and ensure accurate gating thresholds, excluding potential false-positive events due to spectral overlap or intrinsic fluorescence. Following calibration, single-cell suspensions were analyzed using a stepwise gating strategy. Initial gating (left panel) on forward scatter area (FSC-A) versus side scatter area (SSC-A) was used to isolate the main population of intact, single cells while excluding debris and aggregates. The gated population was then evaluated for viability using a fixable Live/Dead UV405 dye (middle panel); live cells, identified by low fluorescence intensity indicating exclusion of the dye, were retained for downstream analysis. Within this live gate, immune cell subsets were identified based on surface marker expression. CD3 and CD56 were used to distinguish T cells ($CD3^{+}CD56^{-}$) from natural killer (NK) cells ($CD3^{-}CD56^{+}$). B cells were identified using dual expression of CD19 and CD45 ($CD19^{+}CD45^{+}$). This sequential gating strategy ensured accurate identification of viable immune subpopulations while minimizing the inclusion of artifacts or non-specific events.

8. Pseudo Code

8.1. Step 1: Define the design factors for the problem, the type of the design factor (continuous, categorical), the number of continuous (Nx) and categorical variables (Nc), the number of categories per categorical variable (C_list/ C), the bounds for the design factors, the type of optimization problem (CoCa vs Co, Unconstrained vs Constrained), Number of initial data points (initN), Number of experiments to be generated in each iteration (batch_size), Measurement noise (Meas_Noise), exploration-exploitation trade-off constant (trade_off)

This information is stored in the dictionary – data_param

Note: Scaling the data to have bounds between [0, 1] is used for numerical stability for the optimizer. The actual upper bound for the design variables is then multiplied - posterior to generate the experimental data.

Step 2: Generate the initial design. If starting from scratch use *design_initial_experiments()* function is used to generate initial data. The jupyter notebooks PBMC_TestFile.ipynb and Kphaffi_TestFile.ipynb illustrates the use of this function and the required inputs.

Step 3: After the designed experiments are performed, data is provided back to the algorithm to generate the next set of experiments using *design_experiments()***.** Provided data must be a concatenation of all previous rounds.

NOTE: In both steps 2 and 3, the background information related to optimization are stored in a .pkl file and preferred filenames are to be provided. This is particularly relevant for categorical continuous optimizations where each iteration's probability distribution and weights are recorded and updated.

8.2. The Analysis_Kphaffi directory has all the Jupyter Notebook files to recreate the plots and includes the sequential data generated during this work.

8.3. The Analysis_PBMC directory has all the Jupyter Notebook and prism files used to analyze and generate the plots and the data generated during the work.

NOTE: It is to be noted that owing to the stochasticity of the approach, the same experiments will likely not be generated every time the code is executed. We have provided the sequential set of data created during this work.
