# Supplementary material for: Accelerating cell culture media development using Bayesian optimization-based iterative experimental design
Source: Nat Commun. 2025 Jul 1;16:6055. doi: 10.1038/s41467-025-61113-5 (PMC12218302; doi:10.1038/s41467-025-61113-5)
Supplement: Supplementary file 2 — Reporting Summary [file 41467_2025_61113_MOESM2_ESM.pdf]

nature portfolio

Corresponding author(s): J Christopher Love

Last updated by author(s): Dec 18, 2024

Reporting Summary

Nature Portfolio wishes to improve the reproducibility of the work that we publish. This form provides structure for consistency and transparency in reporting. For further information on Nature Portfolio policies, see our Editorial Policies and the Editorial Policy Checklist.

Statistics

For all statistical analyses, confirm that the following items are present in the figure legend, table legend, main text, or Methods section.

n/a	Confirmed	
☐	☒	The exact sample size (*n*) for each experimental group/condition, given as a discrete number and unit of measurement
☐	☒	A statement on whether measurements were taken from distinct samples or whether the same sample was measured repeatedly
☐	☒	The statistical test(s) used AND whether they are one- or two-sided<br>*Only common tests should be described solely by name; describe more complex techniques in the Methods section.*
☒	☐	A description of all covariates tested
☒	☐	A description of any assumptions or corrections, such as tests of normality and adjustment for multiple comparisons
☐	☒	A full description of the statistical parameters including central tendency (e.g. means) or other basic estimates (e.g. regression coefficient) AND variation (e.g. standard deviation) or associated estimates of uncertainty (e.g. confidence intervals)
☐	☒	For null hypothesis testing, the test statistic (e.g. *F*, *t*, *r*) with confidence intervals, effect sizes, degrees of freedom and *P* value noted<br>*Give P values as exact values whenever suitable.*
☐	☒	For Bayesian analysis, information on the choice of priors and Markov chain Monte Carlo settings
☒	☐	For hierarchical and complex designs, identification of the appropriate level for tests and full reporting of outcomes
☒	☐	Estimates of effect sizes (e.g. Cohen's *d*, Pearson's *r*), indicating how they were calculated

Our web collection on statistics for biologists contains articles on many of the points above.

Software and code

Policy information about availability of computer code

Data collection	The codes used to generate the experiments, perform the analyses and generate results in this study is publicly available and has been deposited in GitHub at https://github.com/NHarini-1995/CellCultureBayesianOptimization.git, under MIT license. The specific version of the code associated with this publication is archived in Zenodo and is accessible via DOI: 10.5281/zenodo.15466161.
Data analysis	Data was analyzed using python (v 3.7). GPy, GPyOpt and scipy packages were used for modeling and optimizer implementation, respectively. In addition, NumPy, pandas, and matplotlib, seaborn were used for the data reading, processing, analysis and visualization. Jupyter notebooks used for the data analysis is also provided in the GitHub folder.

For manuscripts utilizing custom algorithms or software that are central to the research but not yet described in published literature, software must be made available to editors and reviewers. We strongly encourage code deposition in a community repository (e.g. GitHub). See the Nature Portfolio guidelines for submitting code & software for further information.

Data

Policy information about availability of data

All manuscripts must include a data availability statement. This statement should provide the following information, where applicable:

- Accession codes, unique identifiers, or web links for publicly available datasets
- A description of any restrictions on data availability
- For clinical datasets or third party data, please ensure that the statement adheres to our policy

The media formulations and the corresponding target data that support the findings of this study and were generated in this study are available in figshare with the

identifier(s) doi: https://doi.org/10.6084/m9.figshare.27715134. Source data to create the figures in the paper are provided in DataForFigure.xlsx file.

Research involving human participants, their data, or biological material

Policy information about studies with human participants or human data. See also policy information about sex, gender (identity/presentation), and sexual orientation and race, ethnicity and racism.

Reporting on sex and gender	N/A
Reporting on race, ethnicity, or other socially relevant groupings	N/A
Population characteristics	N/A
Recruitment	N/A
Ethics oversight	N/A

Note that full information on the approval of the study protocol must also be provided in the manuscript.

Field-specific reporting

Please select the one below that is the best fit for your research. If you are not sure, read the appropriate sections before making your selection.

[x] Life sciences   [ ] Behavioural & social sciences   [ ] Ecological, evolutionary & environmental sciences

For a reference copy of the document with all sections, see nature.com/documents/nr-reporting-summary-flat.pdf

Life sciences study design

All studies must disclose on these points even when the disclosure is negative.

Sample size	No particular statistical sample size calculations were performed.<br>For the K.Phaffii use case: A consistency check among replicates were performed in an independent experimental campaign (re-running same experimental condition 6 time). Following that, two biological replicates were performed for the iterative experimental design. A Final campaign was run with best-worst-control experiments in triplicate.<br>For the PBMC use case: Since it is a more noisy system 6 - 8 biological replicates were performed. This was selected to find a trade-off between maximizing biological replicates and other constrains of the experimental system. Particularly, the following factors were considered: (i)maximize the number of replicates given the plate size, (ii) material availability (ii)experimental conditions per iteration, (iii) empty spots at the edges of the plate to avoid any evaporation and (iv) have atleast three biological replicates for viability measurement (since 2 reactions have to be pooled for 1 viability measurement).
Data exclusions	No data was excluded.
Replication	Biological replicates of each experimental condition is included on all the plates. Additionally, a control experiment is also included on each plate. Further more, subset of the conditions from both studies in the work were re-performed in an independent experiment and consistent experimental findings were obtained.
Randomization	PBMC case study: Cell samples in culture plates were placed in random positions on the culture plates, but no other randomization was performed.<br>K.phaffii case study: An independent experimental campaign was performed to study the plate specific effect on the target. Consistency of results were confirmed and no randomization was performed.
Blinding	N/A

Reporting for specific materials, systems and methods

We require information from authors about some types of materials, experimental systems and methods used in many studies. Here, indicate whether each material, system or method listed is relevant to your study. If you are not sure if a list item applies to your research, read the appropriate section before selecting a response.

Materials & experimental systems

n/a	Involved in the study
	☒ Antibodies
	☒ Eukaryotic cell lines
☒	☐ Palaeontology and archaeology
☒	☐ Animals and other organisms
☒	☐ Clinical data
☒	☐ Dual use research of concern
☒	☐ Plants

Methods

n/a	Involved in the study
☒	☐ ChIP-seq
	☒ Flow cytometry
☒	☐ MRI-based neuroimaging

Antibodies

Antibodies used	For flow cytometry in PBMC study: Antibodies were purchased from BioLegend. FITC anti-human CD20 (980202);APC anti-human CD45 982304); APC/Cyanine7 anti-human CD56 (NCAM) Antibody (362512); Zombie Violet (423114).
Validation	Validations were performed by manufacturer. The antibody was purified by affinity chromatography and conjugated with fluorophore under optimal conditions. The confirmed specificity using flow cytometry with standards.

Eukaryotic cell lines

Policy information about cell lines and Sex and Gender in Research

Cell line source(s)	PBMC study: STEMCELL Technologies Catalog # 70025.2<br>K.phaffii study: In-house continuous cell line derived from NRRL Y-11430
Authentication	PBMC cell lines were obtained from standard vendors who performed the authentication.<br>The K.phaffii cell line characterization was performed and reported for the base strain in previous works.
Mycoplasma contamination	All cell lines were tested negative for mycoplasma contamination
Commonly misidentified lines (See ICLAC register)	N/A

Plants

Seed stocks	N/A
Novel plant genotypes	N/A
Authentication	N/A

Flow Cytometry

Plots

Confirm that:

☒ The axis labels state the marker and fluorochrome used (e.g. CD4-FITC).

☒ The axis scales are clearly visible. Include numbers along axes only for bottom left plot of group (a 'group' is an analysis of identical markers).

☒ All plots are contour plots with outliers or pseudocolor plots.

☒ A numerical value for number of cells or percentage (with statistics) is provided.

Methodology

Sample preparation	Peripheral blood mononuclear cells (PBMCs) were isolated from healthy donor blood samples (STEMCELL Technologies) using density gradient centrifugation and pooled into a 1.5 mL Eppendorf tube. A 100 µL aliquot of cells was extracted for viability assessment, while the remaining volume was centrifuged at 500 x g for 8 minutes. During centrifugation, 5 µL of Fc block (Human TruStain FcX; BioLegend) was added to 95 µL of FACS buffer (phosphate-buffered saline [PBS] supplemented with 1%

bovine serum albumin [BSA] and 0.1% sodium azide) per sample. After centrifugation, the supernatant was discarded, and the cells were resuspended in 100 µL of Fc block solution and incubated on ice for 20 minutes. Concurrently, a staining solution was prepared by adding 5 µL of each fluorescent-conjugated antibody (anti-CD20-FITC, anti-CD45-APC, anti-CD56-APC-AF750, Zombie Violet; BioLegend) to FACS buffer, adjusting the volumes according to the number of samples. Cells were incubated on ice in the dark for 30 minutes. Following incubation, the cells were washed twice by centrifugation at 500 x g for 5 minutes and resuspended in 200 µL of FACS buffer.

Instrument	Beckman Coulter CytoFlex LX
Software	FlowJo
Cell population abundance	25 million cells were purchased from STEMCELL Technologies. Once thawed, cell count was checked for purity, and 98% or higher viability vials were used. 50,000 to 100,000 cells were analyzed in each flow experiment, and the variability between the different subsets per experiment is shown in Section 4 of supplementary information
Gating strategy	Flow cytometry gating strategy for cell population identification. The initial gating (left panel) was based on forward and side scatter (FSC-A vs. SSC-A) to isolate the primary cell population. A viability dye (middle panel) was used to distinguish live cells from dead cells, with live cells falling within the defined gate. In the right panels, live cells were further classified into specific immune cell types based on surface markers: NK cells and T cells were identified using CD56 and CD3 markers, while B cells were gated using CD19 and CD45 markers.

☒ Tick this box to confirm that a figure exemplifying the gating strategy is provided in the Supplementary Information.
